# Introduction and Characteristics of SARS-CoV-2 in North-East of Romania During the First COVID-19 Outbreak

**DOI:** 10.3389/fmicb.2021.654417

**Published:** 2021-07-07

**Authors:** Andrei Lobiuc, Mihai Dimian, Roxana Gheorghita, Olga Adriana Caliman Sturdza, Mihai Covasa

**Affiliations:** ^1^Department of Human Health and Development, Stefan cel Mare University of Suceava, Suceava, Romania; ^2^Department of Computers, Electronics and Automation, Stefan cel Mare University of Suceava, Suceava, Romania; ^3^Integrated Center for Research, Development and Innovation in Advanced Materials, Nanotechnologies, and Distributed Systems for Fabrication and Control, Stefan cel Mare University of Suceava, Suceava, Romania; ^4^Regional County Emergency Hospital, Suceava, Romania

**Keywords:** coronavirus, phylogeny, mutations, ORF7, NSP3, routes

## Abstract

Romania officially declared its first Severe Acute Respiratory Syndrome Coronavirus-2 (SARS-CoV-2) case on February 26, 2020. The first and largest coronavirus disease 2019 (COVID-19) outbreak in Romania was recorded in Suceava, North-East region of the country, and originated at the Suceava regional county hospital. Following sheltering-in-place measures, infection rates decreased, only to rise again after relaxation of measures. This study describes the spread of SARS-CoV-2 in Suceava and other parts of Romania and analyses the mutations and their association with clinical manifestation of the disease during the period of COVID-19 outbreak. Sixty-two samples were sequenced *via* high-throughput platform and screened for variants. For selected mutations, putative biological significance was assessed, and their effects on disease severity. Phylogenetic analysis was conducted on Romanian genomes (*n* = 112) and on sequences originating from Europe, United Kingdom, Africa, Asia, South, and North America (*n* = 876). The results indicated multiple introduction events for SARS-CoV-2 in Suceava, mainly from Italy, Spain, United Kingdom, and Russia although some sequences were also related to those from the Czechia, Belgium, and France. Most Suceava genomes contained mutations common to European lineages, such as A20268G, however, approximately 10% of samples were missing such mutations, indicating a possible different arrival route. While overall genome regions ORF1ab, S, and ORF7 were subject to most mutations, several recurring mutations such as A105V were identified, and these were mainly present in severe forms of the disease. Non-synonymous mutations, such as T987N (Thr987Asn in NSP3a domain), associated with changes in a protein responsible for decreasing viral tethering in human host were also present. Patients with diabetes and hypertension exhibited higher risk ratios (RR) of acquiring severe forms of the disease and these were mainly related to A105V mutation. This study identified the arrival routes of SARS-CoV-2 in Romania and revealed potential associations between the SARS-CoV-2 genomic organization circulating in the country and the clinical manifestation of COVID-19 disease.

## Introduction

Coronavirus disease 2019 (COVID-19) emerged in December 2019 in Wuhan City, Hubei Province, China, in humans exposed to wildlife at the Huanan seafood wholesale market (Decaro and Lorusso, 2020). Provisionally named 2019-nCoV, the International Committee on Virus Taxonomy renamed the virus Severe Acute Respiratory Syndrome Coronavirus-2 (SARS-CoV-2) (Lai et al., 2020). Coronaviruses belong to the subfamily Coronavirinae, order Nidovirales and are common human pathogens. They are enveloped, positive-sense RNA viruses with a diameter of 60–140 nm and 29,903 base pair single stranded RNA genome. These viruses are characterized by clublike spike projections of protein on the surface, with a crown-like (from the latin “coronam”) appearance under the electronic microscope (Sharma et al., 2020).

The SARS-CoV-2 colonizes the respiratory tract system causing symptoms similar to those of common cold, such as respiratory disorders, runny nose, dry cough, dizziness, sore throat and body aches, headaches and fever for several days (Mirzaei et al., 2020). In early stages, patients show acute respiratory infection symptoms, with some quickly developing acute respiratory failure and other serious complications (Zheng, 2020). This virus is transmitted from person to person primarily *via* aerosolized droplets (Song et al., 2020). To reduce transmission, preventive measures have been recommended, such as mask wearing, frequent hand washing, limiting contact when symptoms are obvious, avoiding public contact and quarantine (Khailany et al., 2020). Generally, the body’s immune response to SARS-CoV-2 and SARS-CoV is relatively similar and is characterized by an excessive production of cytokines (Astuti and Ysrafil, 2020).

The first whole genome sequence was published on January 5, 2020, and since then thousands of genomes have been sequenced and deposited in the Global Initiative on Sharing All Influenza Data (GISAID) database (van Dorp et al., 2020). This data revealed that it shares approximately 79.6% similarity with SARS-CoV at the nucleotide level and varies between the different genes. SARS-CoV-2 contains a linear single-stranded positive-sense RNA as genetic material that encodes for the spike (S), envelop (E), membrane (M), and nucleocapsid (N) proteins (Gupta and Gupta, 2020). The S glycoprotein is a transmembrane protein found on the viral outer membrane. S protein forms homotrimers that protrude the viral surface and facilitate binding of viral envelope to host cells by interacting with angiotensin-converting enzyme 2 (ACE2) receptors expressed on the lower respiratory tract cells.

Since the introduction of SARS-CoV-2 in territories outside Asia, continuous efforts have been made to map strains and lineages. With time, the viral spread brought about mutations specific to geographical regions, thus making possible to track the virus movement within communities and across the globe. One of the most well-known mutations is in position 23404, changing an aspartate for a glycine at residue 614 in Spike protein and, presumably, offering an advantage in viral replication (Korber et al., 2020). This mutation appeared in January 2020 in China, and after a week in Europe and, was later observed in Africa and Americas (Alouane et al., 2020), giving birth to the “G” clade, now characteristic to Europe (Isabel et al., 2020). In other geographical settings, United States samples share mutations at positions 8782, 17747, 17858, 18060, and 28144, with the first and the latter also present in European samples. Such signatures, composed of multiple recurrent mutations within the same region have been considered, when identifying founder effects for that lineage (Farkas et al., 2020).

Identifying mutations and strains movement across geographical regions is critical for predicting further infection hotspots, as well as for vaccine and diagnostic tests development. This can be obtained by sequencing and analyzing the complete viral genomes, thus allowing a comprehensive view of all genetic variants at once. This is particularly important as biological effects, including the ability of the virus to evade detection or immunity, can be induced by single or concurrent mutations, while tracking the viral evolution and spread can be effectively monitored. Sequencing data adds significant resolution to regular molecular testing and can aid in medical prognostic, informed medical decisions, allowing epidemiologists to perform more directed epidemiological enquiries.

The first patient with COVID-19 in Romania was confirmed on February 26, 2020, in Gorj county, South-West of Romania (Streinu-Cercel, 2020). On March 3, 2020, patient number 6 was diagnosed with COVID-19 and hospitalized at the Regional County Hospital of Suceava, North East of Romania. This led to a rapid contamination of medical personnel and the Regional Hospital of Suceava became the largest outbreak of COVID-19 in the country that still leads in the number of confirmed cases and deaths nationwide. Suceava Regional Hospital serves more than 600,000 people and is the largest hospital in the North-East region of the country. On March 26, a SARS-CoV-2 diagnostic laboratory (RT-PCR based) was set up within the hospital that allowed identification of patients with SARS-CoV-2 infection. Suceava county has one of the largest migrant population working in EU countries who begun returning to Romania once COVID-19 spread across Europe. The initial epidemiological analysis on a small sample of patients (*n* = 147) from Bucharest (capital of Romania) and several counties collected between February and March 2020 indicated that Romanian migrants from Italy were the main introduction routes of virus spread (Hâncean et al., 2020). In order to understand the introduction and transmission of the virus that led to this largest outbreak, we performed sequencing and phylogenetic analyses of samples from patients with confirmed SARS-CoV-2 infection from Suceava county. We then compared the data with those reported from other regions of the country as well as several European countries, Asia, Africa and America continents accessed from GISAID data. Finally, we examined whether specific variants in SARS-CoV-2 proteins were associated with patients’ clinical parameters and disease outcomes. The results of this study will aid in uncovering the routes of introduction of the virus that lead to the first and largest outbreak in Romania and points to particular mutations, of potential biological and epidemiological interest.

## Materials and Methods

### Sample Collection

Viral RNA was obtained from samples of patients hospitalized in the Suceava County Regional Hospital, collected between 10.04.2020 and 19.06.2020. Patients signed informed consent for data access and the study was approved by the University of Suceava Research Ethics Committee (protocol number 11733/14.07.2020). Criteria for patient selection included age, sex, severity of the disease, number of days in hospital and existing comorbidities. Samples were collected by nasopharyngeal swabs from patients presenting with COVID-19-like symptoms. Clinical, epidemiological and demographic data were taken from patients’ medical records.

### Sample Preparation and Sequencing

RNA extraction was performed using Bioneer AccuPrep^®^ Viral RNA Extraction Kit. RNA extracts were evaluated for viral copy numbers (TaqMan 2019-nCoV Assay Kit v1, Applied Biosystems, United States), and SARS-CoV-2 positive samples were selected for analysis, based on the numbers of the viral copies as well as on RNA quantity for each sample. RNA (100 ng) was reverse transcribed using SuperScript^TM^ VILO^TM^ cDNA Synthesis Kit (Invitrogen, United States), according to product protocol. Targets for sequencing were obtained based on Ion AmpliSeq^TM^ SARS-CoV-2 Panel (ThermoFisher, United States). Library preparation was made using Ion AmpliSeq^TM^ Library Kit Plus (ThermoFisher, United States), then libraries were loaded on sequencing chips using Ion Chef equipment. Next generation sequencing was performed on Ion S5 Gene Studio, using Ion Torrent 540 chips.

### Sequencing Data Processing and Data Availability

Sequencing reads were mapped and assembled using the Iterative Refinement Meta Assembler (IRMA), after which variants were called with Torrent VariantCaller plugin, referenced to the Wuhan SARS-CoV-2 sequence and annotated using SnpEff plugin. Sequences were uploaded in GISAID database on 2020-07-11 and 2020-07-16.

### Phylogenetic Analysis

To assess phylogenetic placement of Romanian viral samples that included those from Suceava and other regions, a GISAID survey was performed, selecting European, United Kingdom, Africa, Asia, South and North America genomes, under the “high coverage,” “low coverage excluded,” “complete” criteria. All 112 Romanian samples present in GISAID at the time of writing this article were included, together with another 1,043 sequences from the aforementioned continents. The accessions were selected to include representative genomes from each lineage. After removing identical sequences from the same geographical region, and those with long stretches of “*nnn*,” a total of 876 samples were considered for analyses. Prior to phylogenetic analyses, all samples were aligned using MAFFT algorithm, then trimmed at ends (first 50 nucleotides and last 80 nucleotides), to remove unnecessary artifacts caused by sequencing in those areas. Since mutations were recorded in almost all genomic regions, all phylogenetic analyses were conducted using the entire sequences, not only specific regions, to allow tracking of all mutations with epidemiological significance.

Phylogenetic analysis of our sequenced samples and selected European ones was performed based on maximum likelihood algorithms, using RaxML-HPC2 workflow on CIPRES platform, phylo.org (Miller et al., 2010). In order to select a nucleotide substitution model, preliminary analyses were carried out using jModelTest on CIPRES. Set parameters were GTR Gamma model and a bootstrap value of 1,000. The resulting tree was loaded, visualized and annotated in ITOL platform, itol.embl.de (Letunic and Bork, 2019). Bayesian time dated phylogenetic analysis of the data set was performed using BEAST 2.6.3, with Beagle library enabled. A HKY + Γ model of nucleotide substitution and a strict clock were assigned, using a coalescent exponential population model. A continuous-time Markov chain was employed, posterior distributions of parameters were estimated by sampling every 5,000 steps over a total of 50 million MCMC steps. Each analysis was run in duplicate to check for convergence, and the first 10% of samples were discarded as burn-in. Sampling was considered effective when a minimum of 200 Estimated Sample Size was reached.

### Statistical Analyses

Differences between patient groups (asymptomatic, mild, severe) were assessed using ANOVA, followed by a Duncan *post hoc* test, while correlations were evaluated by Spearman’s coefficient. Ordinal or binary logistic regression was applied for disease severity categories, while binary logistic regression and risk ratio (RR) estimation were applied when various factors (sex, comorbidities) were assessed in relation with patient’s status. All statistical analyses were conducted using Minitab 19.2020.1 (Minitab LLC, PA, United States). Nucleotide modifications (replacements and indels) were tabulated and frequencies per disease severity groups were calculated. The number of transitions and transversions and their ratios were calculated per entire genome and per each gene.

## Results

### Characteristics of Sequenced Samples From Suceava

**FIGURE 1:**
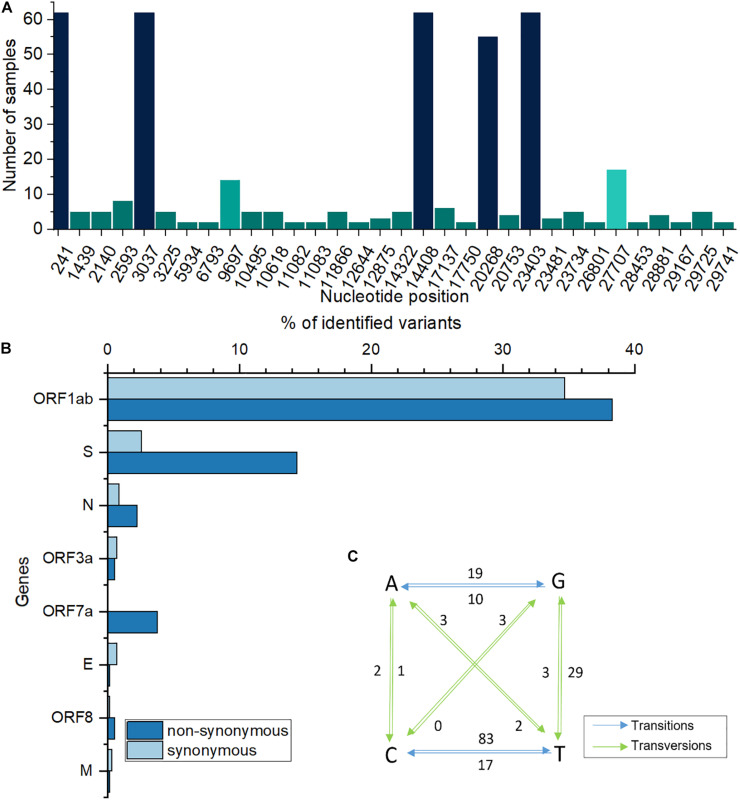
**(A)** Most frequently modified nucleotide positions and corresponding number of samples. **(B)**. Percentages of genome-wide synonymous/non-synonymous variants. **(C)** Base changes in analyzed sequences. Numbers next to arrows indicate how many times a particular base change was recorded; T is replaced by U in the original, RNA sequence; blue, transitions; green, transversions.

A total of 62 samples were selected, based on quality checks, comprising 39 samples from males and 23 from females. When compared with Wuhan reference Genome, GISAID accession ID Nc_045512.2, a total of 190 modifications were recorded and distributed across eight genome regions. With ORF1ab being the largest SARS-CoV-2 gene (approximately 24 kb), corresponding to a polyprotein made up of 16 non-structural proteins (NSP1-16), over 66% of all mutations were recorded in this region. This was followed by the spike (S) and nucleocapsid (N) protein coding genes while other genes, such as ORF3a, ORF7a or envelope (E) represented less than 5% of all mutations. When observing instances of individual mutations, rather than gene-wise frequencies, most frequent modified nucleotides were recorded at positions 241, 3037, 14408, 23403, 20268, 27707, and 9697, totaling 57% of all modifications. These mutations were present in as few as 5, and up to all 62 samples. A distribution of recurrent modifications throughout the genome (∼30 kb) is shown in Figure 1A. Of the mutations recorded, the highest proportion belongs to transitions, accounting for over 76% of them, while only 23.7% are transversions. Specifically, C to T (U) transitions make up almost 50% of all SNPs recorded, followed by G to T (U) transversions (17%), A to G (11%) and T (U) to C (10%) transitions (Figure 1C). Overall, the transitions: transversions ratio is 3.2:1, with a value > 1 for most genes.

When considering amino acid sequences, modifications at most frequent positions were of synonymous type, however, a large proportion induced amino acid alterations, such as those at positions 314 in ORF7a and 1841 in S genes (Table 1). At the entire genome level, for certain ORFs, the number of non-synonymous mutations was higher than that of synonymous ones (Figure 1B). The well-known S-protein mutation, 614 D > G that gives rise to GISAID “G” clade, was present in all 62 samples. Other key signatures of SARS-CoV-2 evolution were changes at positions 14408 and 20268.

**TABLE 1:** Frequent nucleotide and aminoacid modifications in analyzed genomes.

**Nucleotide position**	**Genome region**	**Position within gene/Nucleotide modification**	**Aminoacid modification**	**Type of modification**	**Frequency**
241	Orf1ab	c.-25C > T	Non-coding	Upstream genevariant	100%
3225	ORF1ab	2960C > A	p.Thr987Asn	Non-synonymous variant	27.41%
23403	S	1841A > G	p.Asp614Gly	missense_variant	100%
20268	ORF1ab	20003A > G	p.Ter6668Trpext?	stop_lost	88.70%
27707	ORF7a	314C > T	p.Ala105Val	missense_variant	27.41%

A notable feature was the mutation at nucleotide position 27707, leading to a change from alanine to valine in ORF7a sequence, at aminoacid 105 (A105V) and present in 17 out of the 62 analyzed samples. Another recurring mutation is represented by a change from cytosine to adenine at position 3225, leading to a substitution of threonine by asparagine at position 987 (T987N) of the ORF1ab region. The majority of nucleotide modifications preserved the reading frames of the genetic sequences, however, a number of deletions led to gene variants, including frameshift ones (Table 2). Out of these, a modification, in position 29725, in the 3′ untranslated region (3′ UTR), was present in five out of 62 analyzed samples (8%).

**TABLE 2:** Nucleotide modifications leading to gene variants.

**Nucleotide position**	**Genome reference**	**Type of modification**	**Reference sequence**	**Modification**
509	ORF1ab	Frameshift variant	GGTCATGTTAT GGTT	G
1597	ORF1ab	Conservative inframe deletion	AGGTCTT	A
4861	ORF1ab	Frameshift variant	TAA	T
5817	ORF1ab	Frameshift variant	CT	C
7434	ORF1ab	Conservative inframe deletion	TTTA	T
8925	ORF1ab	Frameshift variant	GA	G
11082	ORF1ab	Frameshift variant	TG	T
14407	ORF1ab	Frameshift variant	CCTACAAG	C
14723	ORF1ab	Frameshift variant	TC	T
18058	ORF1ab	Disruptive inframe deletion	CTCT	C
19827	ORF1ab	Frameshift variant	GA	G
22844	S	Frameshift variant	GATTTTACA GGCTGCGTT	G
25159	S	Frameshift variant	TC	T
25826	ORF3a	Conservative inframe deletion	ATTT	A
29725	3′UTR	Downstream gene variant deletion	AT	A
29727	3′UTR	Downstream gene variant deletion	TTTCACCGA GGCCACGCG GAGTACGAT CGAGTGTAC AGTG	T
29755	3′UTR	Downstream gene variant deletion	GAGTGTAC	G

### Phylogenetic Analyses

**FIGURE 2:**
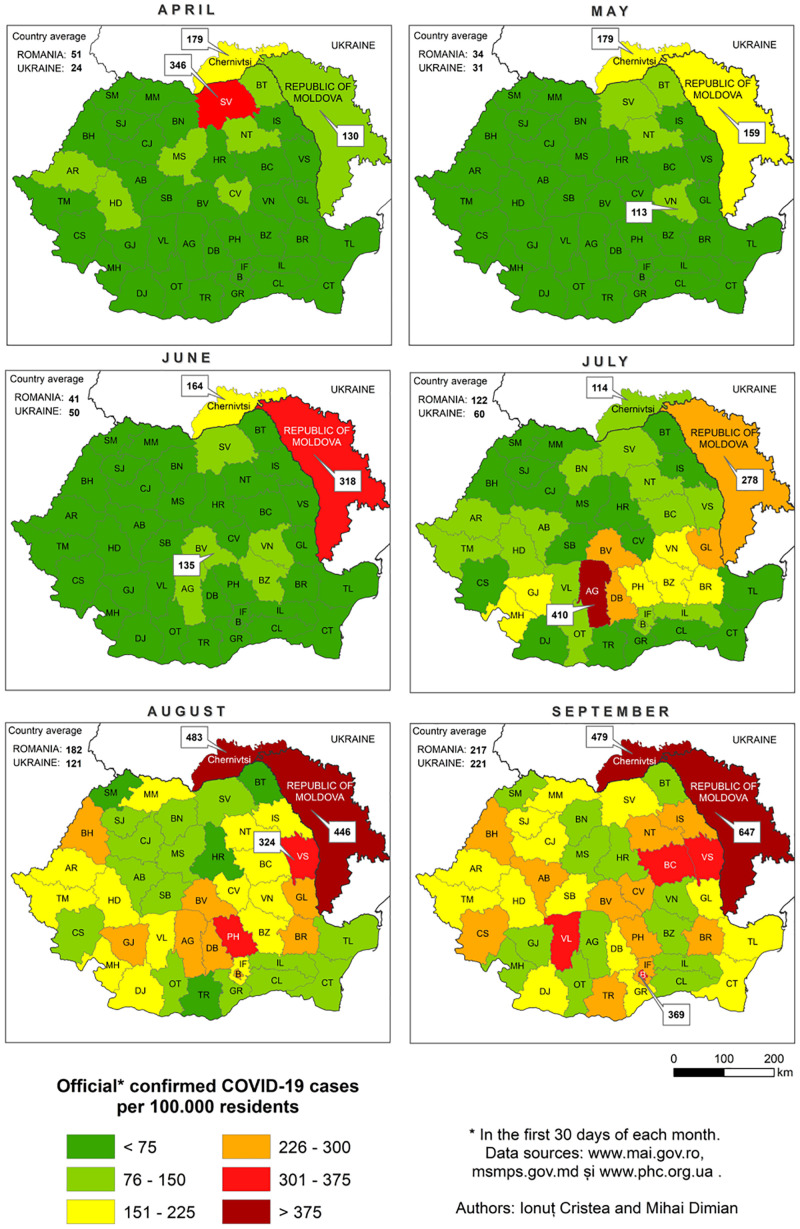
The evolution of SARS-CoV-2 infections in Suceava (SV) and the rest of Romania, during April-September 2020.

**FIGURE 3:**
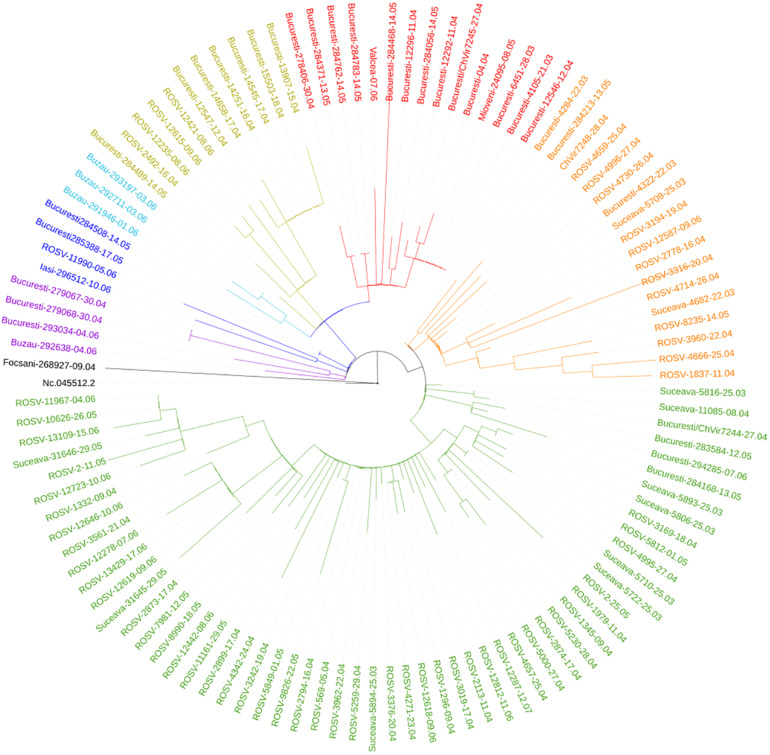
Phylogenetic distribution of Romanian genomes (*n* = 112).

**FIGURE 4:**
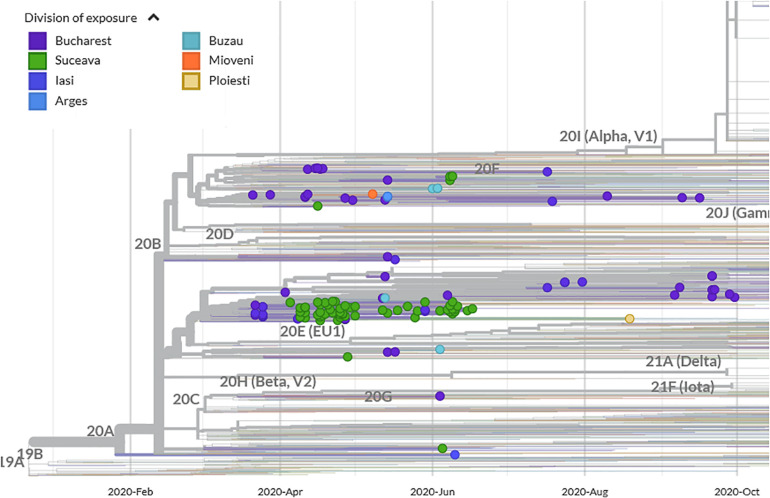
Phylogenetic analysis of SARS-CoV-2 emergence in Suceava and other Romanian regions within Nextstrain phylogeny/clades (showing genomes as filtered by Nextstrain visualization platform).

Suceava outbreak was the first and the largest in Romania. The evolution of SARS-CoV-2 infection was chronologically different in Suceava, compared to other regions of the country, peaking in March then decreasing sharply while in the rest of the country, particularly in the North East region, there was a gradual increase in case prevalence (Figure 2). To examine the phylogenetic distribution of SARS-CoV-2 from Suceava county (North East of Romania), we first compared these samples (*n* = 62) with those from other regions of Romania (*n* = 50). All sequences were submitted to GISAID^1^ with accession numbers listed in Supplementary Table 1. The total number of samples (*n* = 112) was grouped in six clusters, four of which included samples from Suceava. Most Suceava samples (*n* = 45) were grouped in a large cluster, together with few genomes from Bucharest (South of Romania). A smaller number of samples sequenced in our laboratory (*n* = 12) belonged to another distinct cluster with samples from Bucharest. Four samples clustered with those from Bucharest and Buzau (South East of Romania) and one sample was closer to a sample sequenced from Iasi (North East of Romania) (Figure 3). We then compared phylogenetic distribution of Romanian samples with representative European viral genomes. To do this, a maximum likelihood tree was constructed, using bootstrapped RaxML (Supplementary Figure 1). Overall, Romanian viral genomes formed several clusters with those from European regions such as Spain, Russia, Italy, Turkey, England and Austria (Supplementary Figure 1). Importantly, the Romanian phylogenetic clustering was mostly preserved in the larger European phylogenetic analysis. There was a distinct geographical and temporal genomic organization. As such, genomes from the North East region of the country (Suceava and Iasi) were grouped mainly with genomes from Spain, Italy, Egypt, United Kingdom, and Russia. Most Suceava sequences from the early period of the pandemic (March to mid-April) were related to Spanish and Italian genomes as well as to an Egyptian genome, while those from the later months (mid-April to June), were related to Russian and United Kingdom genomes. In addition, several sequences from Suceava were grouped with those from the Czechia. In the Southern region of the country (Bucharest and Mioveni), samples from the early periods were associated with genomes from Spain, Italy, and Turkey, while those from later periods, were associated with genomes from France, Belgium, and Russia. Also, some genomes from the Southern region (Buzau), from June were closer to those from the United Kingdom. Approximately one third of the Suceava genomes, from May and June, were associated with one genome from Egypt (Africa), which belonged to the GISAID G clade. No associations were detected between Romanian genomes and those from Asia or American continents. Thus, the possible routes of introduction of SARS-CoV-2 in Romania, observed when inferring phylogenies using Nextstrain based on more than 30.000 GISAID sequences support the relationship between Romanian genomes and the genomes from the countries mentioned above. When considering GISAID pangolin lineages, most of our sequenced genomes from Suceava (*n* = 57) belong to the B.1.5 lineage, GISAID G clade, while four belong to the B.1.1 lineage, GR clade and one to the B.1, G clade. When taking into account the European samples subset provided by Nextstrain, 28 of our samples from Suceava belong to 20A clade, while two belong to the 20B clade (Figure 4). Moreover, one cluster from Suceava is formed based on the mutation at position 27707 (A105V), in the ORF7a region. The temporal dynamic within analyzed samples was confirmed through a Bayesian inference of genomes, in order to calculate mean time to most recent ancestor (tMRCA). The date obtained was November 2019, using both HKY and GTR + gamma substitution model, confirming the temporal signal (0.954 correlation coefficient, 639 Estimated Sample Size – ESS) present in our sequenced samples.

### Clinical Parameters and Outcomes

Of the 62 patients from whom viral sequences were obtained, 23 were females, and 39 were males (average 52.4 years). With respect to the severity of the disease, six patients were asymptomatic, 35 had a mild condition, and 21 displayed a severe form. The following clinical criteria were used for defining disease severity: mild/moderate illness: individuals who had any of the various signs and symptoms of COVID-19 (e.g., fever, cough, sore throat, malaise, headache, muscle pain, nausea, vomiting, diarrhea, loss of taste, and smell) but who do not have shortness of breath, dyspnea, or abnormal chest imaging. It includes individuals with evidence of lower respiratory disease during clinical assessment or imaging and an oxygen saturation (SpO_2_) ≥ 94% on room air. Severe illness included individuals with SpO_2_ < 94% on room air, a ratio of arterial partial pressure of oxygen to fraction of inspired oxygen (PaO_2_/FiO_2_) < 300 mm Hg, respiratory frequency > 30 breaths/min, or lung infiltrates > 50%, rapid decrease of lymphocytes and increase of lactate dehydrogenase or patients with ARDS, septic shock, or organ failure requiring intensive care.

The hospitalization length ranged between 4 and 39 days (average 20 days). In order to identify factors that significantly influenced disease severity and clinical outcome we examined viral copy number, number of mutations, number of hospitalized days, age, sex and certain comorbidities, such as diabetes, obesity and hypertension. Among continuous variables, only the number of hospitalized days showed significant differences between asymptomatic and mild status patients (Table 3). Although the average number of hospitalized days was highest in mildly diseased patients, the hospitalization days ranged from 4 to 22 days for asymptomatic, 7–39 days for mild status and 9–34 days for severe status. Sex was not significantly correlated with the severity of disease (Pearson’s rho = 0.17).

**TABLE 3:** Sample and patient related factors.

**Factor**		**Asymptomatic**	**Mild**	**Severe**
Viral copies		6.05E + 04^*a*^ ± 5.23E + 04	8.86E + 04^*a*^ ± 2.49E + 04	8.40E + 04^*a*^ ± 3.49E + 04
Hospitalized days		12.40^*a*^ ± 3.43	23.39^*b*^ ± 1.76	18.41^*ab*^ ± 1.91
Age		57.83^*a*^ ± 3.98	49.80^*a*^ ± 3.47	55.10^*a*^ ± 3.79
Comorbidities (%)	Hypertension	–	34.28	38.09
	Obesity	16.67	31.42	28.57
	Diabetes	–	14.28	19.04

*Superscripts with different letters on the same row indicate statistical significance (*p* < 0.05).*

When examining the effect of comorbidities on clinical outcome, the percentages of hypertensive, diabetic and obese patients were higher among mild and severe forms (Table 3). When comparing asymptomatic patients with mild and severe forms, calculated RR for hypertension and diabetes were 1.58 (C.I. 95% 0.8–3.1) and 1.9 (C.I. 95% 0.58–6.1), respectively. None of the above factors (viral copy number, days of hospitalization, and comorbidities) were significantly associated with the number of deaths which may have been due to the low numbers of deceased patients (*n* = 7). It should be noted however, that out of the seven deceased patients, two had hypertension, one was diabetic, and three were obese.

**FIGURE 5:**
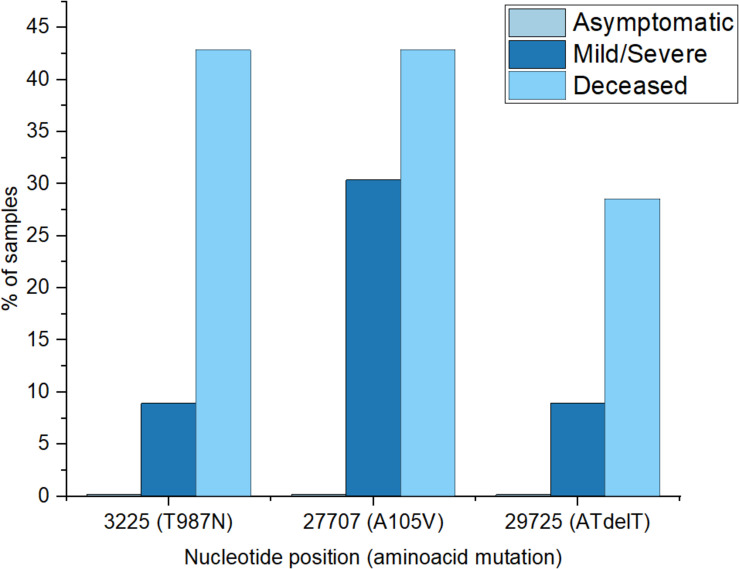
Percentages of frequent recurrent mutations encountered in different patient groups (asymptomatic group registered none of the selected mutations).

To investigate possible effects of viral mutations on disease outcome, we screened for non-synonymous recurring mutations, in patients with asymptomatic, mild/moderate and severe clinical manifestation as well as in those deceased. We found that 30% of the mild/severe cases had the A105V mutation in the ORF7a region and 8.9% presented a T987N mutation in NSP3 domain, ORF1ab region. The same recurring mutations were identified in the deceased patients, with 42.8% presenting the A105V mutation, 42.8% the T987N mutations and 28.5%, the nucleotide deletion at position 29725 (Figure 5). There was no significant effect of age or sex on the presence of mutations. Patients infected with the virus carrying the A105V mutation had a significant increase in C-Reactive Protein (9.66 ± 2.7 mg/dL) compared to the wild type (5.45 ± 1.14 mg/dL), *P* = 0.039.

**FIGURE 6:**
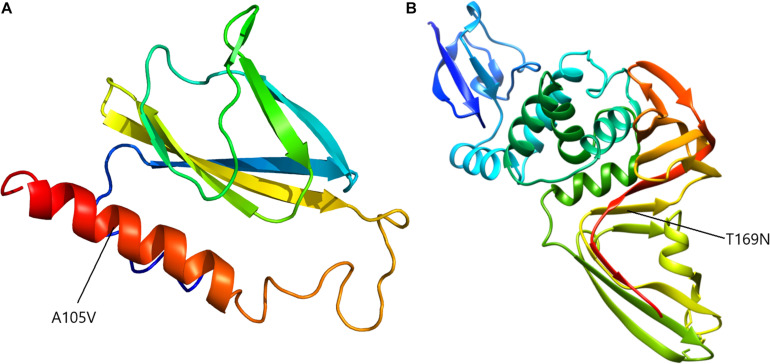
*Ab initio* model of an entire ORF7a protein **(A)** and NSP3a domain **(B)**.

To assess the putative effect of mutations on viral structure, proteins corresponding to mutated regions were modeled accordingly. Mutation at position 27707 (C > T) altered the ORF7a protein, 121 aminoacid residues long, changing the alanine in position 105 to a valine. Considering that analytical structural data is only available for the 16–82 residues range, protein modeling was performed *ab initio*, independently, using Phyre2 (Kelley et al., 2015) and I-TASSER (Zhang, 2008), for both mutated and wild proteins (Figure 6A). Superimposing structures yielded a RMSD value of 2.33 Å for Phyre2 predicted structures and 2.29 Å for I-TASSER ones and, correspondingly, a TM score of 0.85 for both cases.

The second mutation considered for modeling was T987N in ORF1ab. This mutation appeared in the papain-like protease (NSP3) coding region, at aminoacid residue number 169, domain NSP3a. This region is described mainly for SARS-CoV-1 and consists of two subdomains, a ubiquitin-like domain (Ubl1) and a Glu-rich, acidic region, located at the N-terminal of NSP3. For SARS-CoV-2, no computational protein models are available for this region, thus, after alignment with the similar domain of SARS-CoV-1, the first 205 residues of NSP3 were *ab initio* modeled. The two models, for mutated and original wild-type sequence, recorded a RMSD of 3.81 Å and a TM-score of 0.60 (Figure 6).

## Discussion

The sequence and phylogenetic analyses of SARS-CoV-2 genomes showed that Romanian samples appear heterogenous in the quality and quantity of mutations and supports the idea of multiple viral introductory events in the country. When integrating sequencing data with clinical parameters of patients, our results identified specific viral mutations that were associated with the degree of disease severity and patients’ comorbidities.

### Characteristics and Introduction of SARS-CoV-2 in Romania

Among detected single nucleotide polymorphisms, there was a high, 3:1 ratio of transitions to transversions. This ratio was described in SARS-CoV-2 mutations, where, supposedly, cytosine deaminases may be responsible for C > T changes, while G > T ones may be the result of oxo-guanine arising from reactive oxygen species (Koyama et al., 2020). The SNP profile of our sequenced samples is in line with the large picture observed in over 40,000 worldwide samples regarding the prevalence of C > T mutations (Mercatelli and Giorgi, 2020). However, secondly abundant in our samples, was the G > T transversion, as compared to A > G transition, which ranks third. This higher C > T numbers might be explained by the differential activity of viral RNA editing enzymes. For example, APOBECs (Apolipoprotein B mRNA editing complex) and ADARs (Adenosine Deaminases Acting on RNA), generate such mutations on positive strand RNA, pointing to the abundance of this form or viral RNA, over negative strand RNA within samples (Mishra et al., 2020). The rates of transitions to transversions greater than 1, indicate possible high incidence of non-synonymous variants. Such nucleotide modifications may lead to replacement of hydrophilic aminoacids with hydrophobic ones, which can significantly change protein properties.

All 62 samples analyzed from Suceava share a set of mutation common to European samples. Specifically, mutations at positions 241 (non-coding), 3037 C > T, 14408 C > T, 20268 20003A > G, and 23403 A > G are frequent in European samples (Alouane et al., 2020) and were identified early in the pandemic evolution, as a signature for one of the superspreaders that originated from Wuhan (Mishra et al., 2020). Among the most abundant observed mutations, 241 C > T belongs to the leader sequence, with significance for discontinuous sub-genomic replication. This mutation co-evolved with other three major mutations, 3037 C > T, 14408 C > T, and 23403 A > G. As a consequence, a synonymous mutation in NSP3, a P323L mutation in RNA primase, and a mutation in spike glycoprotein (614 D > G) occurred. The combination of these four mutations was mostly observed in European genomes and is associated with more severe forms of infection and, possibly, with increased transmissibility (Yin, 2020). Interestingly, the signature of another super spreader, which was less encountered in European genomes, namely the change in 11083 G > T was present in two of our samples. This mutation was observed in samples from France and China (Yang et al., 2020).

Mutation 20268 A > G was recorded in samples collected before 26 of May, in 45% of samples from Spain (Liu et al., 2020) and was also present in most of our samples from Suceava. However, seven of our 62 sequenced samples did not share this mutation, including those belonging to B.1 and B.1.1 lineages but not the B.1.5. The genome belonging to B.1 lineage, sampled on June 5, was close in our phylogenetic analysis, with another Romanian sample from Iasi (North East of Romania) and, overall, with samples from France and Belgium. Several B.1.1 lineage samples, were grouped, along with samples from Russia (Moscow, St. Petersburg, Novosibirsk), comprising early (April) samples from both Southern Romania (Bucharest) and Northern part of the country (Suceava). A third B.1.1 lineage sample, from June 8 was closer to samples from Turkey. When examining sampling dates for sequences from Suceava without 20268 A > G mutation, it appears that they were introduced in Suceava around April 16, possibly from the Czechia.

When examining Romanian sequences belonging to B.1.1 our data show that B1.1 lineage was introduced in Romania approximately late March to early April primarily from England and Italy, and, possibly Russia. When we examined the global distribution of the genomes, Romanian samples were grouped only with those from Europe, United Kingdom, Russia and with one genome from Africa (Egypt), while none were associated with the genome from Asia or American continents. Romanian samples belonging to B.1.5 lineage were mixed, mainly with samples from Russia, Italy, and Spain. These results support multiple introduction events of the virus in the country, with one route being Spain, Italy, and Turkey, the second England and Czechia and a third from East Russia and beyond.

The introductory events of SARS-CoV-2 in Romania have been influenced by migration, restriction measures and specific religious events. At the beginning of the pandemic over 800,000 Romanian migrants returned to Romania of which approximately 10,000 returned to Suceava and became significant vectors of transmission (Lucheş et al., 2021) resulting in the first and largest outbreak of COVID-19 in the country. A set of restrictions imposed by the government were enforced on March 30, 2020, thus further transmission in Suceava was mainly intracommunitary. This is supported by our findings showing that until April 15, the genomes sequenced were associated mainly with those from Italy and Spain where a large part of Romanian diaspora is concentrated. Epidemiological data also show that the largest proportion of cases was imported from Italy (Hâncean et al., 2020). After the Orthodox Easter on April 18, 2020 that coincided with another wave of migrants and subsequent rise in COVID-19 cases, the sequences analyzed were mainly associated with those from the United Kingdom genome.

### COVID-19 and Patient Status

Examination of COVID-19 patient data from Suceava showed a high variability in clinical manifestation and disease severity. Our study showed that comorbidities such as hypertension and diabetes may be associated with more severe forms of the disease, which is in line with previous work as well as with results from a recent study in Romanian COVID-19 patients indicating RR with values of 6.4 for diabetes and 3.3 for hypertension (Barbu et al., 2020; Mazucanti and Egan, 2020; Schiffrin et al., 2020). Currently, there is not a consensus on the mechanisms by which hypertension or diabetes increase morbidity risk in COVID-19 patients. ACE-1 inhibitors and angiotensin II receptor blockers, that are used for diabetics and hypertensives, upregulate ACE-2 expression, the SARS-CoV-2 receptor. While this may have protective effects against lung injury, it increases the chances of acquiring the disease. Likewise, a hyperglycemic environment increases virulence of some pathogens, phagocytosis, chemotaxis, response of T cells, and neutrophils, decreased immune response while production of interleukins is restricted (Cristelo et al., 2020). Furthermore, coronaviruses may increase glycemic levels by damaging pancreatic islet cells (Yang et al., 2010). ACE-2 expression polymorphism present in humans could influence both the susceptibility and outcome of COVID-19 (Devaux et al., 2020). Finally, in our patients, age was not related with disease severity, outcome or length of hospitalization, although age was considered a risk factor in Italy (Poletti et al., 2020) and might be a driver for disease trajectory (Ayoub et al., 2020). Our data showed higher viral load in samples from mild as compared to severe COVID-19 cases. This was likely due to the time of sample collection from onset of infection that varied between patients. It should be also noted that in as much as some studies have linked viral load with disease severity and mortality, there is no great correlation between how much virus is detected and increased morbidity. In fact, several studies showed severe COVID-19 cases unrelated to high viral titers, and by contrast, individuals with high viral loads that are relatively asymptomatic (Argyropoulous et al., 2020).

### Viral Protein Modifications

Among mutations in SARS-CoV-2, some result in protein structure and properties changes. One such mutation is 614 D > G, which, over the course of 1 month, became prevalent in viral strains worldwide. Although 614 D > G mutation was associated with lower Ct values in RT-PCR analyses (Korber et al., 2020), our samples recorded a very large range of Cts, between 17 and 36, suggesting that several factors, probably both virus and host related, influence viral titers. This mutation is associated with less shedding of S1 subdomain of the S protein, increased viral stability and transmission, although not necessarily with increased disease severity (Zhang et al., 2020), while other studies associated 614 D > G and 4715 P > L with increased mortality.

Another non-synonymous recurrent mutation in a large number of our samples was A105V in ORF7a. Our model of protein modification showed that this mutation has a high probability to modify an alfa-helix stretch into a beta-sheet conformer since valine is a hydrophobic amino acid known to be one of the best β-sheet former (Caillet-Boudin et al., 1991). Therefore, the replacement of alanine 105 residue to valine favors β-sheet‘s secondary structure. The ORF7a region in genomes of coronaviruses encodes a 5.5 kDa protein, with a putative role in enhancing virulence in SARS-CoV. In SARS-CoV-1, the 122 residues long protein (accessory protein 7a) and is an integral membrane protein, and localizes in the Golgi compartments, probably in the budding regions (Nelson et al., 2005). It was shown to be involved in cell apoptosis, through caspase-dependent pathway, cell protein synthesis inhibition, cell cycle progression blockage and proinflammatory action, thus altering the host cellular environment (Yuan et al., 2006; Schaecher and Pekosz, 2010). Interestingly, this protein is known to interact with a viral release inhibitor, bone marrow stromal antigen 2 (BST-2 or CD317 or tetherin). BST-2 is an interferon-inducible factor that tethers various nascent enveloped viruses on the cell surface, playing an important role in viral infection (Mahauad-Fernandez et al., 2014). The antiviral function of BST-2, in the case of SARS-CoV, occurs by stopping the egression of virions through the plasma membrane and inhibits glycosylation of BST-2 while removes its antiviral function (Taylor et al., 2015). Although ORF7a was proposed as a potential candidate for antiviral drug development (Almasi and Mohammadipanah, 2020), other groups have reported significant mutations in ORF7a (Holland et al., 2020).

The increased occurrence of this mutation in COVID-19 patients, but not in asymptomatic ones suggests its role in triggering a more intense inflammatory response. For example, C-reactive protein was shown to be a marker for disease severity, even the in early stages of COVID-19 (Wang, 2020). As such, patients infected with A105V had higher values of CRP, compared with non-A105V infected patients. The increase in inflammation is associated with acute respiratory distress, distributive shock, myocardial injury, and hemodynamic changes (Reyes et al., 2020). Further, induction of the innate immune response is dependent on interferon (IFN) stimulation. Coronaviruses, including SARS-CoV-2, evade IFN based responses, by expressing open reading frames, such as ORF7a, thus abolishing IFN pathways (Salman et al., 2021). As such, a more stable ORF7, due to a A105V mutation, may reduce immune response and, consequently, more severe COVID symptoms. Although a clear clinical effect of ORF7a mutation cannot be predicted, they are expected to result in decreased antigen-presenting ability and much higher expression of pro-inflammatory cytokines (Zhou et al., 2021). At the time of writing the paper, this mutation was reported in another 78 samples on the CovGLUE platform (Singer et al., 2020) that was located mainly in the United Kingdom, but also in Denmark, Netherlands, Germany, United States, and Australia.

Another recurrent mutation in our samples associated with a large proportion of severe disease cases, was the T987N replacement in ORF1ab polyprotein. This mutation occurs in the structurally disordered domain, involves the amino acid residue 169 of the NSP3a domain and constitutes in a Thr → Asn substitution. NSP3a is one of the seven domains of SARS-CoV NSP3 polypeptide known to be involved in RNA replication (Snijder et al., 2003) and consists of a 112-residue N-terminal subdomain with a homogeneous content of amino acids and a C-terminal subdomain rich in acidic residues. NMR studies revealed that the subdomain NSP3a (aminoacids 1–112) exhibits a globular ubiquitin-like fold with two additional helices while the Glu-rich acidic domain (residues 113–183), also called “hypervariable region,” was shown to be structurally disordered (Serrano et al., 2007). These unique structural elements are involved in interactions with single-stranded RNA.

The NSP3 domain appears to be also involved in affecting IFN pathways induction and increased inflammatory host response. Apparently, viral macrodomains such as NSP3 affects ADP-ribosylation, an important process in efficient immune response, by binding ADP-ribose (Alhmmad and Fehr, 2020). Meanwhile, NSP3 has a great variability in coronaviruses and might induce specific clinical manifestations of the associated diseases, with increased cytokine storm and, consequently, more severe inflammation (Claverie, 2020), which can explain the occurrence of this mutation in severe forms of the disease, as opposed to asymptomatic forms. This mutation has been reported in only 10 samples, according to CoV-GLUE database and the literature is scarce on its effect on clinical outcomes.

Structural similarities with proteins involved in various cell-signaling pathways indicate possible roles of NSP3a in viral infection and persistence. The function of the glutamic acid-rich region is still not known; however, similar Glu-rich region was observed in the transcription factor Mytl1l known to be involved in the general function of binding nucleic acids. NSP3a can be classified as a “low complexity region,” found in many viruses, including Coronaviridae. Such regions are considered to be highly immunogenic and, importantly, they share high similarity with human epitopes, thus placing them a risk for antiviral drug or testing development (Gruca et al., 2020). The enrichment of glutamic acid was found as a feature of the highly immunogenic polypeptides, in other organisms as well (Hou et al., 2020).

## Conclusion

Coronavirus disease 2019 became an ubiquitary presence in Romania, affecting all groups of individuals regardless of age, sex or other factors. The SARS-CoV-2 arrival routes that triggered the first largest outbreak of COVID-19 in Romania appear to be multiple. Viral genomes identified in the North East region of the country were primarily related to those from Italy and Spain during the early phase and to those from the United Kingdom during the later phase of the outbreak. Fewer genomes were also related to those from the Czechia, Russia, Turkey, Belgium, and France. Specific mutations in the regions ORF1a and ORF7 were identified that were associated with the severity of the disease. Most patients infected with the virus containing the specific ORF7 A105V mutation presented severe forms of the disease, including increased inflammatory markers. Also, comorbidities such as hypertension and diabetes likely contributed to the severe manifestation of COVID-19. The detection of mutations that may lead to severe forms of the diseases requires constant monitoring and integration of clinical data with genome sequencing results. Nevertheless, our study revealed potential associations between the SARS-CoV-2 genomic organization circulating in Romania, routes of introduction to the country and the identification of risk factors both in the virus and in the host that could contribute to the progression of the natural history of infection by this virus. Further viral genomic analyses evolution is critical for detection of mutations, virus containment and timely treatment.

## Data Availability Statement

The datasets presented in this study can be found in online repositories. The names of the repository/repositories and accession number(s) can be found in the article/Supplementary Material.

## Ethics Statement

The studies involving human participants were reviewed and approved by Stefan cel Mare University of Romania. The patients/participants provided their written informed consent to participate in this study.

## Author Contributions

AL, MC, and MD designed the study. AL and RG performed the experimental work. OACS and RG collected the data. AL, RG, MD, and MC analyzed the data. AL and MC wrote the first draft. All authors read and approved the final manuscript.

## Conflict of Interest

The authors declare that the research was conducted in the absence of any commercial or financial relationships that could be construed as a potential conflict of interest.

## Footnotes

1 https://www.gisaid.org/

## Supplementary Material

Supplementary Figure 1 Phylogenetic distribution of SARS-CoV-2 genome sequences from Romania in relation with genomes worldwide. Legend indicates clusters with bootstrap support including Romanian (RO) genomes and genomes from other countries/territories.

Supplementary Table 1 Accession number of Romanian/Suceava SARS-CoV-2 genome sequences submitted to GISAID.

## Abbreviations

Asn: Asparagine
GISAID: GISAID EpiFlu ^TM^ Database and Data,https://www.gisaid.org/
IL-8: Interleukin 8
kb: kilobases
MAP: mitogen activated protein kinase
NF- κ B, Nuclear factor: kappa B
NSP3a, Non-structural protein 3: subdomain 3a
ORF: Open reading frame
Thr: Threonine.

## References

[B1] Alhmmad Y. Fehr A. R. (2020). The Viral Macrodomain Counters Host Antiviral ADP-Ribosylation. *Viruses* 12:384. 10.3390/v12040384 32244383 PMC7232374

[B2] Almasi F. Mohammadipanah F. (2020). Hypothetical targets and plausible drugs of coronavirus infection caused by SARS-CoV-2. *Transbound. Emerg. Dis.* 68 318–332. 10.1111/tbed.13734 32662203 PMC7405402

[B3] Alouane T. Laamarti M. Essabbar A. Hakmi M. Bouricha E. M. Chemao-Elfihri M. W. (2020). Genomic diversity and hotspot mutations in 30,983 SARS-CoV-2 genomes: moving toward a universal vaccine for the “confined virus”? *bioRxiv* [Preprint]. 10.1101/2020.06.20.163188 PMC7600297 33050463

[B4] Argyropoulous K. Serrano A. Hu J. Black M. Feng X. Shen G. (2020). Association of initial viral load in severe acute respiratory syndrome coronavirus 2 (SARS-CoV-2) patients with outcome and symptoms. *Am. J. Pathol.* 190 1881–1887. 10.1016/j.ajpath.2020.07.001 32628931 PMC7332909

[B5] Astuti I. Ysrafil (2020). Severe Acute Respiratory Syndrome Coronavirus 2 (SARS-CoV-2): an overview of viral structure and host response. *Diabetes Metab. Syndr.* 14 407–412. 10.1016/j.dsx.2020.04.020 32335367 PMC7165108

[B6] Ayoub H. H. Chemaitelly H. Seedat S. Mumtaz G. R. Makhoul M. Abu-Raddad L. J. (2020). Age could be driving variable SARS-CoV-2 epidemic trajectories worldwide. *medRxiv* [Preprint]. 10.1101/2020.04.13.20059253 PMC7444586 32817662

[B7] Barbu M. G. Thompson R. J. Thompson D. C. Cretoiu D. Suciu N. (2020). The Impact of SARS-CoV-2 on the Most Common Comorbidities–A Retrospective Study on 814 COVID-19 Deaths in Romania. *Front. Med.* 7:567199. 10.3389/fmed.2020.567199 33015111 PMC7509043

[B8] Caillet-Boudin M. L. Lemay P. Boulanger P. (1991). Functional and structural effects of an Ala to Val mutation in the adenovirus serotype 2 fibre. *J. Mol. Biol.* 217 477–486. 10.1016/0022-2836(91)90751-q 1994035

[B9] Claverie J. M. A. (2020). A Putative Role of de-Mono-ADP-Ribosylation of STAT1 by the SARS-CoV-2 Nsp3 Protein in the Cytokine Storm Syndrome of COVID-19. *Viruses* 12:646. 10.3390/v12060646 32549200 PMC7354589

[B10] Cristelo C. Azevedo C. Marques J. M. Nunes R. Sarmento B. S. A. R. S. - (2020). CoV-2 and diabetes: new challenges for the disease. *Diabetes Res. Clin. Pract.* 164:108228. 10.1016/j.diabres.2020.108228 32446801 PMC7242186

[B11] Decaro N. Lorusso A. (2020). Novel human coronavirus (SARS-CoV-2): a lesson from animal coronaviruses. *Vet. Microbiol.* 244:108693. 10.1016/j.vetmic.2020.108693 32402329 PMC7195271

[B12] Devaux C. A. Rolain J. M. Raoult D. (2020). ACE2 receptor polymorphism: susceptibility to SARS-CoV-2, hypertension, multi-organ failure, and COVID-19 disease outcome. *J. Microbiol. Immunol. Infect.* 53 425–435. 10.1016/j.jmii.2020.04.015 32414646 PMC7201239

[B13] Farkas C. Fuentes-Villalobos F. Garrido J. L. Haigh J. Barría M. I. (2020). Insights on early mutational events in SARS-CoV-2 virus reveal founder effects across geographical regions. *PeerJ* 8:e9255. 10.7717/peerj.9255 32509472 PMC7246029

[B14] Gruca A. Ziemska-Legiecka J. Jarnot P. Sarnowska E. Sarnowski T. J. Grynberg M. (2020). Common low complexity regions for SARS-CoV-2 and human proteomes as potential multidirectional risk factor in vaccine development. *bioRxiv* [Preprint]. 10.1101/2020.08.11.245993 PMC8027979 33832440

[B15] Gupta T. Gupta S. K. (2020). Potential adjuvants for the development of a SARS-CoV-2 vaccine based on experimental results from similar coronaviruses. *Int. Immunopharmacol.* 86:106717. 10.1016/j.intimp.2020.106717 32585611 PMC7301105

[B16] Hâncean M.-G. Perc M. Lerner J. (2020). Early spread of COVID-19 in Romania: imported cases from Italy and human-to-human transmission networks. *R. Soc. Open Sci.* 7:200780. 10.1098/rsos.200780 32874663 PMC7428275

[B17] Holland L. A. Kaelin E. A. Maqsood R. Estifanos B. Wu L. I. Varsani A. (2020). An 81 base-pair deletion in SARS-CoV-2 ORF7a identified from sentinel surveillance in Arizona. *medRxiv* [Preprint]. 10.1128/JVI.00711-20 PMC7343219 32357959

[B18] Hou N. Jiang N. Ma Y. Zou Y. Piao X. Liu S. (2020). Low-complexity repetitive epitopes of Plasmodium falciparum are decoys for humoural immune responses. *Front. Immunol.* 11:610. 10.3389/fimmu.2020.00610 32351503 PMC7174639

[B19] Isabel S. Graña-Miraglia L. Gutierrez J. M. Bundalovic-Torma C. Groves H. E. Isabel M. R. (2020). Evolutionary and structural analyses of SARS-CoV-2 614 D>G spike protein mutation now documented worldwide. *Sci Rep.* 10:14031. 10.1038/s41598-020-70827-z PMC7441380 32820179

[B20] Kelley L. A. Mezulis S. Yates C. M. Wass M. N. Sternberg M. J. E. (2015). The Phyre2 web portal for protein modeling, prediction and analysis. *Nat. Protoc.* 10 845–858. 10.1038/nprot.2015.053 25950237 PMC5298202

[B21] Khailany R. A. Safdar M. Ozaslan M. (2020). Genomic characterization of a novel SARS-CoV-2. *Gene Rep.* 19:100682. 10.1016/j.genrep.2020.100682 32300673 PMC7161481

[B22] Korber B. Fischer W. M. Gnanakaran S. Yoon H. Theiler J. Abfalterer W. (2020). Tracking Changes in SARS-CoV-2 Spike: evidence that 614 D>G Increases Infectivity of the COVID-19 Virus. *Cell* 182 812–827.e19. 32697968 10.1016/j.cell.2020.06.043 PMC7332439

[B23] Koyama T. Platt D. Parida L. (2020). Variant analysis of SARS-cov-2 genomes. *Bull World Health Organ.* 98 495–504. 32742035 10.2471/BLT.20.253591 PMC7375210

[B24] Lai C. C. Shih T. P. Ko W. C. Tang H. J. Hsueh P. R. (2020). Severe acute respiratory syndrome coronavirus 2 (SARS-CoV-2) and coronavirus disease-2019 (COVID-19): the epidemic and the challenges. *Int. J. Antimicrob. Agents* 55:105924. 10.1016/j.ijantimicag.2020.105924 PMC7127800 32081636

[B25] Letunic I. Bork P. (2019). Interactive Tree of Life (iTOL) v4: recent updates and new developments. *Nucleic Acids Res.* 47 W256–W259. 30931475 10.1093/nar/gkz239 PMC6602468

[B26] Liu S. Shen J. Fang S. Li K. Liu J. Yang L. (2020). Genetic spectrum and distinct evolution patterns of SARS-CoV-2. *Front. Microbiol.* 11:593548. 10.3389/fmicb.2020.593548 33101264 PMC7545136

[B27] Luche D. Saghin D. Lupchian M. M. (2021). Public perception of the first major SARS-Cov-2 outbreak in the Suceava County, Romania. *Int. J. Environ. Res. Public Health* 18:1406. 10.3390/ijerph18041406 33546326 PMC7913496

[B28] Mahauad-Fernandez W. D. Jones P. H. Okeoma C. M. (2014). Critical role for bone marrow stromal antigen 2 in acute Chikungunya virus infection. *J. Gen. Virol.* 95 2450–2461. 10.1099/vir.0.068643-0 25053563 PMC4202266

[B29] Mazucanti C. H. Egan J. M. (2020). SARS-CoV-2 disease severity and diabetes: why the connection and what is to be done? *Immun. Ageing* 17:21. 10.1186/s12979-020-00192-y PMC7325192 32612666

[B30] Mercatelli D. Giorgi F. M. (2020). Geographic and Genomic Distribution of SARS-CoV-2 Mutations. *Front. Microbiol.* 11:1800. 10.3389/fmicb.2020.01800 32793182 PMC7387429

[B31] Miller M. A. Pfeiffer W. Schwartz T. (2010). “Creating the CIPRES Science Gateway for inference of large phylogenetic trees,” in *2010 Gateway Computing Environments Workshop, GCE 2010*, (New York: IEEE).

[B32] Mirzaei R. Mohammadzadeh R. Mahdavi F. Badrzadeh F. Kazemi S. Ebrahimi M. (2020). Overview of the current promising approaches for the development of an effective severe acute respiratory syndrome coronavirus 2 (SARS-CoV-2) vaccine. *Int. Immunopharmacol.* 88:106928. 10.1016/j.intimp.2020.106928 32862110 PMC7444935

[B33] Mishra A. Kumar Pandey A. Gupta P. Pradhan P. Dhamija S. Gomes J. (2020). Mutation landscape of SARS-CoV-2 reveals three mutually exclusive clusters of leading and trailing single nucleotide substitutions. *bioRxiv* [Preprint]. 10.1101/2020.05.07.082768

[B34] Nelson C. A. Pekosz A. Lee C. A. Diamond M. S. Fremont D. H. (2005). Structure and intracellular targeting of the SARS-coronavirus orf7a accessory protein. *Structure* 13 75–85. 10.1016/j.str.2004.10.010 15642263 PMC7125549

[B35] Poletti P. Tirani M. Cereda D. Trentini F. Guzzetta G. Marziano V. (2020). Age-specific SARS-CoV-2 infection fatality ratio and associated risk factors, Italy, February to April 2020. *Eurosurveillance* 25:2001383. 10.2807/1560-7917.ES.2020.25.31.2001383 PMC7459272 32762797

[B36] Reyes A. Z. Hu A. K. Teperman J. Muskardin T. Tardif J. Shah B. (2020). Anti-inflammatory therapy for COVID-19 infection: the case for colchicine. *Ann. Rheum. Dis.* 80 550–557. 10.1136/annrheumdis-2020-219174 33293273 PMC8491433

[B37] Salman A. A. Waheed M. H. Ali-A bdulsahib A. A. Atwan Z. W. (2021). Low type I interferon response in COVID-19 patients: interferon response may be a potential treatment for COVID-19. *Biomed. Rep.* 14:43. 10.3892/br.2021.1419 PMC7995242 33786172

[B38] Schaecher S. R. Pekosz A. (2010). “SARS coronavirus accessory gene expression and function,” in *Molecular Biology of the SARS-Coronavirus*, ed. Sunil Lal K. (Berlin: Springer), 153–166. 10.1007/978-3-642-03683-5_10

[B39] Schiffrin E. L. Flack J. M. Ito S. Muntner P. Webb R. C. (2020). Hypertension and COVID-19. *Am. J. Hypertens.* 33 373–374. 32251498 10.1093/ajh/hpaa057 PMC7184512

[B40] Serrano P. Johnson M. A. Almeida M. S. Horst R. Herrmann T. Joseph J. S. (2007). Nuclear Magnetic Resonance Structure of the N-Terminal Domain of Nonstructural Protein 3 from the Severe Acute Respiratory Syndrome Coronavirus. *J. Virol.* 81 12049–12060. 10.1128/jvi.00969-07 17728234 PMC2168779

[B41] Sharma A. Tiwari S. Deb M. K. Marty J. L. (2020). Severe acute respiratory syndrome coronavirus-2 (SARS-CoV-2): a global pandemic and treatment strategies. *Int. J. Antimicrob. Agents* 56:106054. 10.1016/j.ijantimicag.2020.106054 PMC7286265 32534188

[B42] Singer J. Gifford R. J. Cotten M. Robertson R. L. (2020). CoV-GLUE: a web application for tracking sars-cov-2 genomic variation. *Preprints* 10.20944/preprints202006.0225.v1 32283112

[B43] Snijder E. J. Bredenbeek P. J. Dobbe J. C. Thiel V. Ziebuhr J. Poon L. L. M. (2003). Unique and conserved features of genome and proteome of SARS-coronavirus, an early split-off from the coronavirus group 2 lineage. *J. Mol. Biol.* 331 991–1004. 10.1016/s0022-2836(03)00865-9 12927536 PMC7159028

[B44] Song P. Li W. Xie J. Hou Y. You C. (2020). Cytokine storm induced by SARS-CoV-2. *Clin. Chim. Acta* 509 280–287. 32531256 10.1016/j.cca.2020.06.017 PMC7283076

[B45] Streinu-Cercel A. (2020). Sars-Cov-2 in Romania – situation update and containment strategies. *GERMS* 10:8. 10.18683/germs.2020.1179 32274354 PMC7117882

[B46] Taylor J. K. Coleman C. M. Postel S. Sisk J. M. Bernbaum J. G. Venkataraman T. (2015). Severe Acute Respiratory Syndrome Coronavirus ORF7a Inhibits Bone Marrow Stromal Antigen 2 Virion Tethering through a Novel Mechanism of Glycosylation Interference. *J. Virol.* 89 11820–11833. 10.1128/jvi.02274-15 26378163 PMC4645327

[B47] van Dorp L. Acman M. Richard D. Shaw L. P. Ford C. E. Ormond L. (2020). Emergence of genomic diversity and recurrent mutations in SARS-CoV-2. *Infect. Genet. Evol.* 83:104351. 10.1016/j.meegid.2020.104351 32387564 PMC7199730

[B48] Wang L. (2020). C-reactive protein levels in the early stage of COVID-19. *Med. Mal. Infect.* 50 332–334. 10.1016/j.medmal.2020.03.007 32243911 PMC7146693

[B49] Yang J. K. Lin S. S. Ji X. J. Guo L. M. (2010). Binding of SARS coronavirus to its receptor damages islets and causes acute diabetes. *Acta Diabetol.* 47 193–199. 10.1007/s00592-009-0109-4 19333547 PMC7088164

[B50] Yang X. Dong N. Chan E. W. C. Chen S. (2020). Genetic cluster analysis of SARS-CoV-2 and the identification of those responsible for the major outbreaks in various countries. *Emerg. Microbes Infect.* 9 1287–1299. 10.1080/22221751.2020.1773745 32525765 PMC7477621

[B51] Yin C. (2020). Genotyping coronavirus SARS-CoV-2: methods and implications. *Genomics* 112 3588–3596. 10.1016/j.ygeno.2020.04.016 32353474 PMC7184998

[B52] Yuan X. Wu J. Shan Y. Yao Z. Dong B. Chen B. (2006). SARS coronavirus 7a protein blocks cell cycle progression at G0/G1 phase via the cyclin D3/pRb pathway. *Virology* 346 74–85. 10.1016/j.virol.2005.10.015 16303160 PMC7111786

[B53] Zhang L. Jackson C. Mou H. Ojha A. Rangarajan E. Izard T. (2020). The 614 D>G mutation in the SARS-CoV-2 spike protein reduces S1 shedding and increases infectivity. *bioRxiv* [Preprint]. 10.1101/2020.06.12.148726 32587973 PMC7310631

[B54] Zhang Y. (2008). I-TASSER server for protein 3D structure prediction. *BMC Bioinformatics* 9:40. 10.1186/1471-2105-9-40 18215316 PMC2245901

[B55] Zheng J. (2020). SARS-coV-2: an emerging coronavirus that causes a global threat. *Int. J. Biol. Sci.* 16 1678–1685. 10.7150/ijbs.45053 32226285 PMC7098030

[B56] Zhou Z. Huang C. Zhou Z. Huang Z. Su L. Kang S. (2021). Structural insight reveals SARS-CoV-2 Orf7a as an immunomodulating factor for human CD14+ monocytes. *iScience* 24:102187. 10.1016/j.isci.2021.102187 33615195 PMC7879101

