# Supplementary figures and images for: Introduction and Characteristics of SARS-CoV-2 in North-East of Romania During the First COVID-19 Outbreak
Source: Front Microbiol. 2021 Jul 7;12:654417. doi: 10.3389/fmicb.2021.654417 (PMC8292954; doi:10.3389/fmicb.2021.654417)

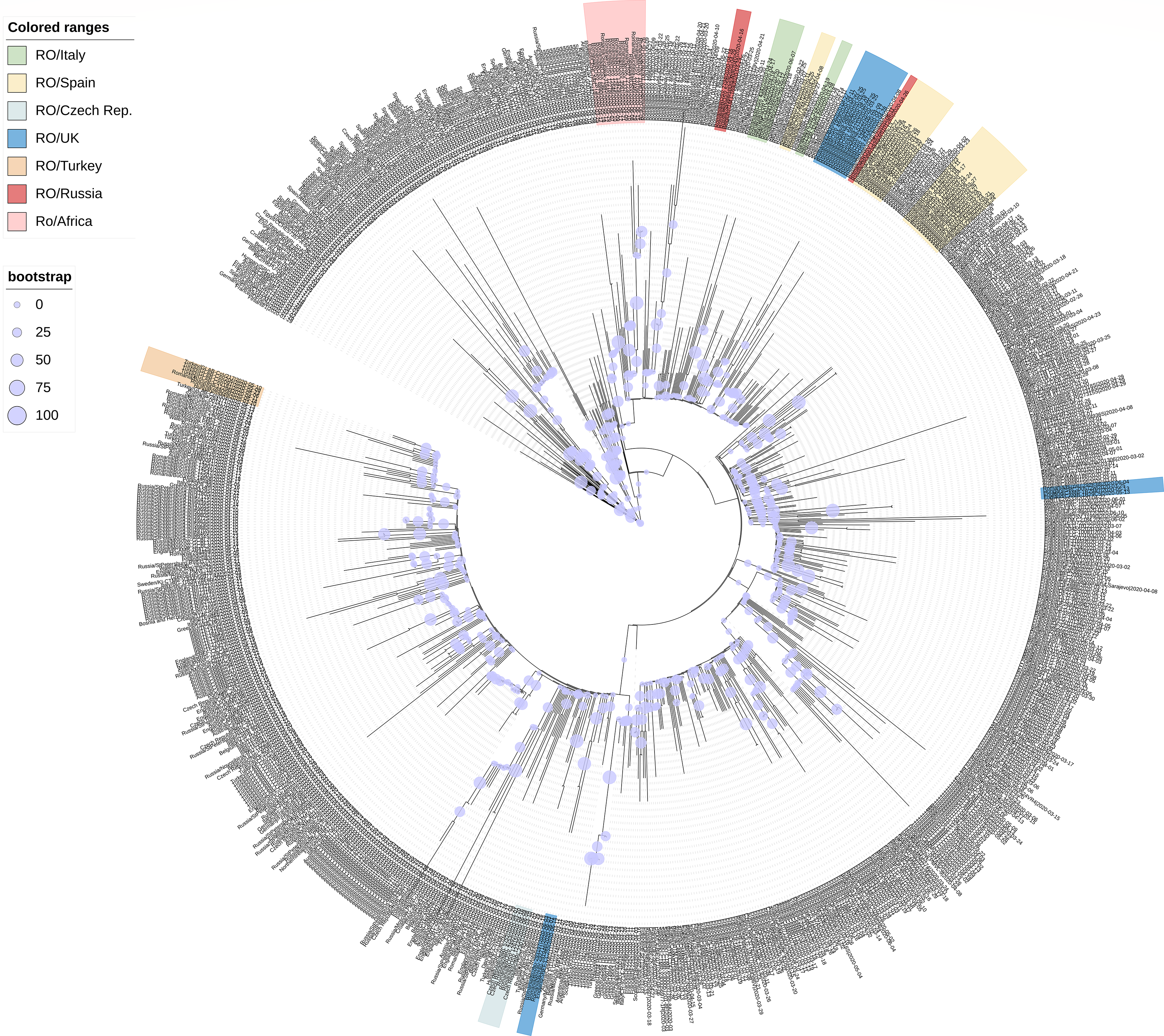

Supplement: Supplementary Figure 1 — Phylogenetic distribution of SARS-CoV-2 genome sequences from Romania in relation with genomes worldwide. Legend indicates clusters with bootstrap support including Romanian (RO) genomes and genomes from other countries/territories. [file Image_1.TIFF]
