# Supplementary material for: Introduction and Characteristics of SARS-CoV-2 in North-East of Romania During the First COVID-19 Outbreak
Source: Front Microbiol. 2021 Jul 7;12:654417. doi: 10.3389/fmicb.2021.654417 (PMC8292954; doi:10.3389/fmicb.2021.654417)
Supplement: Supplementary Table 1 — Accession number of Romanian/Suceava SARS-CoV-2 genome sequences submitted to GISAID. [file Data_Sheet_1.PDF]

S1 Table. Accession number of Romanian/Suceava SARS-CoV-2 genome sequences submitted to GISAID

No.	Virus name	Accession ID	Collection date
1	hCoV-19/Romania/Suceava-4682/2020	EPI_ISL_468137	22/03/2020
2	hCoV-19/Romania/Suceava-5709/2020	EPI_ISL_468138	25/03/2020
3	hCoV-19/Romania/Suceava-5710/2020	EPI_ISL_468139	25/03/2020
4	hCoV-19/Romania/Suceava-5722/2020	EPI_ISL_468140	25/03/2020
5	hCoV-19/Romania/Suceava-5806/2020	EPI_ISL_468141	25/03/2020
6	hCoV-19/Romania/Suceava-5816/2020	EPI_ISL_468142	25/03/2020
7	hCoV-19/Romania/Suceava-5893/2020	EPI_ISL_468143	25/03/2020
8	hCoV-19/Romania/Suceava-5894/2020	EPI_ISL_468144	25/03/2020
9	hCoV-19/Romania/Suceava-11085/2020	EPI_ISL_468146	08/04/2020
10	hCoV-19/Romania/Suceava-31645/2020	EPI_ISL_468157	29/05/2020
11	hCoV-19/Romania/Suceava-31646/2020	EPI_ISL_468158	29/05/2020
12	hCoV-19/Romania/Iasi-296512/2020	EPI_ISL_471421	10/06/2020
13	hCoV-19/Romania/ROSV-2/2020	EPI_ISL_486834	25/05/2020
14	hCoV-19/Romania/ROSV_12646/2020	EPI_ISL_486854	10/06/2020
15	hCoV-19/Romania/ROSV/2020	EPI_ISL_486855	08/06/2020
16	hCoV-19/Romania/ROSV_12287/2020	EPI_ISL_486856	2020
17	hCoV-19/Romania/ROSV_10626/2020	EPI_ISL_491036	26/05/2020
18	hCoV-19/Romania/ROSV_11161/2020	EPI_ISL_491037	29/05/2020
19	hCoV-19/Romania/ROSV_11967/2020	EPI_ISL_491038	04/06/2020
20	hCoV-19/Romania/ROSV_11990/2020	EPI_ISL_491039	05/06/2020
21	hCoV-19/Romania/ROSV_12235/2020	EPI_ISL_491040	08/06/2020
22	hCoV-19/Romania/ROSV_12278/2020	EPI_ISL_491041	07/06/2020
23	hCoV-19/Romania/ROSV_12421/2020	EPI_ISL_491042	08/06/2020
24	hCoV-19/Romania/ROSV_12442/2020	EPI_ISL_491043	08/06/2020
25	hCoV-19/Romania/ROSV_12587/2020	EPI_ISL_491044	09/06/2020
26	hCoV-19/Romania/ROSV_12615/2020	EPI_ISL_491045	09/06/2020
27	hCoV-19/Romania/ROSV_12618/2020	EPI_ISL_491046	09/06/2020
28	hCoV-19/Romania/ROSV_12619/2020	EPI_ISL_491047	09/06/2020
29	hCoV-19/Romania/ROSV_12723/2020	EPI_ISL_491048	10/06/2020
30	hCoV-19/Romania/ROSV_12812/2020	EPI_ISL_491049	11/06/2020
31	hCoV-19/Romania/ROSV_1296/2020	EPI_ISL_491050	09/04/2020
32	hCoV-19/Romania/ROSV_13109/2020	EPI_ISL_491051	15/06/2020
33	hCoV-19/Romania/ROSV_1332/2020	EPI_ISL_491052	09/04/2020
34	hCoV-19/Romania/ROSV_13429/2020	EPI_ISL_491053	17/06/2020
35	hCoV-19/Romania/ROSV_1345/2020	EPI_ISL_491054	09/04/2020
36	hCoV-19/Romania/ROSV_1837/2020	EPI_ISL_491055	11/04/2020
37	hCoV-19/Romania/ROSV_1979/2020	EPI_ISL_491056	11/04/2020
38	hCoV-19/Romania/ROSV_2113/2020	EPI_ISL_491057	11/04/2020
39	hCoV-19/Romania/ROSV_2492/2020	EPI_ISL_491058	16/04/2020
40	hCoV-19/Romania/ROSV_2778/2020	EPI_ISL_491059	16/04/2020
41	hCoV-19/Romania/ROSV_2794/2020	EPI_ISL_491060	16/04/2020
42	hCoV-19/Romania/ROSV_2873/2020	EPI_ISL_491061	17/04/2020
43	hCoV-19/Romania/ROSV_2874/2020	EPI_ISL_491062	17/04/2020
44	hCoV-19/Romania/ROSV_2899/2020	EPI_ISL_491063	17/04/2020
45	hCoV-19/Romania/ROSV_3019/2020	EPI_ISL_491064	17/04/2020
46	hCoV-19/Romania/ROSV_3169/2020	EPI_ISL_491065	18/04/2020

47	hCoV-19/Romania/ROSV_3194/2020	EPI_ISL_491066	19/04/2020
48	hCoV-19/Romania/ROSV_3242/2020	EPI_ISL_491067	19/04/2020
49	hCoV-19/Romania/ROSV_3316/2020	EPI_ISL_491068	20/04/2020
50	hCoV-19/Romania/ROSV_3376/2020	EPI_ISL_491069	20/04/2020
51	hCoV-19/Romania/ROSV_3561/2020	EPI_ISL_491070	21/04/2020
52	hCoV-19/Romania/ROSV_3960/2020	EPI_ISL_491071	22/04/2020
53	hCoV-19/Romania/ROSV_3962/2020	EPI_ISL_491072	22/04/2020
54	hCoV-19/Romania/ROSV_4271/2020	EPI_ISL_491073	23/04/2020
55	hCoV-19/Romania/ROSV_4342/2020	EPI_ISL_491074	24/04/2020
56	hCoV-19/Romania/ROSV_4657/2020	EPI_ISL_491075	25/04/2020
57	hCoV-19/Romania/ROSV_4659/2020	EPI_ISL_491076	25/04/2020
58	hCoV-19/Romania/ROSV_4666/2020	EPI_ISL_491077	25/04/2020
59	hCoV-19/Romania/ROSV_4714/2020	EPI_ISL_491078	26/04/2020
60	hCoV-19/Romania/ROSV_4730/2020	EPI_ISL_491079	26/04/2020
61	hCoV-19/Romania/ROSV-1815/2020	EPI_ISL_678398	11/04/2020
62	hCoV-19/Romania/ROSV-10281/2020	EPI_ISL_678397	25/04/2020
